# Scrotal migration of inguinal hernia repair mesh: an unusual cause of testicular mass

**DOI:** 10.1259/bjrcr.20150427

**Published:** 2016-05-08

**Authors:** Gabriele Maffi, Lorenzo Carlo Pescatori, Giovanni Mauri, Luca Maria Sconfienza

**Affiliations:** ^1^Scuola di Specializzazione in Radiodiagnostica, Università degli Studi di Milano, Milano, Italy; ^2^Dipartimento di Radiologia Diagnostica ed Interventistica, Istituto Europeo di Oncologia, Milano, Italy; ^3^Servizio di Radiologia, IRCCS Policlinico San Donato, Milano, Italy; ^4^Dipartimento di Scienze Biomediche per la Salute, Università degli Studi di Milano, Milano, Italy

## Abstract

Scrotal masses are common findings in primary care and can develop from all anatomical structures of the scrotum. They are usually painless, although pain may be present occasionally. In this report, we present the case of an unusual testicular mass caused by the migration in the scrotum of a mesh used for inguinal hernia repair. The patient was treated conservatively owing to the spontaneous resolution of symptoms.

## Summary

Scrotal masses are common findings in primary care and can develop from all anatomical structures of the scrotum such as testes, epididymis, appendix of the epididymis, appendix of the testes, gubernaculum testis, vas deferens, pampiniform plexus and spermatic cord.^1^ Most paratesticular masses are benign and only require intervention if they enlarge or cause pain. The most frequent are cysts of the epididymis that occur in up to 40% of men. Primary malignancies of the epididymis or paratesticular structures are extremely rare in adults.^2^ Metastases could also be found occasionally.^3^

Patients presenting with acute scrotal pain to the emergency department represent less than 1% of all the cases and require a timely diagnosis.

In this report, we present the case of an unusual testicular mass seen at the emergency department of our institution in a patient with previous scrotal pain.

## Clinical presentation

A 60-year-old male was admitted to the emergency department of our hospital for a palpable mass in the scrotum. The patient was sent by the general practitioner for suspected testicular torsion, as he reported an onset of sudden intense pain over the left side of the scrotum.

On admission to the hospital, a few hours after having been seen by the general practitioner, the patient reported a complete resolution of pain. He denied symptoms of the urinary tract. He reported a clinical history of left inguinal hernia repair about 30 years ago, with the implantation of a mesh. However, no clinical documents were brought for reference. He recently underwent mitral valvuloplasty and was on oral anticoagulant therapy.

On physical examination, a tender palpable mass cranial/superficial to the epididymis was found. There were no signs of acute inflammation and no pain was elicited on local or abdominal palpation. Vital signs were good: heart rate was 80 beats min^–1^, blood pressure was 120/75 mmHg and oxygen saturation was 100%.

## Investigations/imaging findings

**Figure 1.**
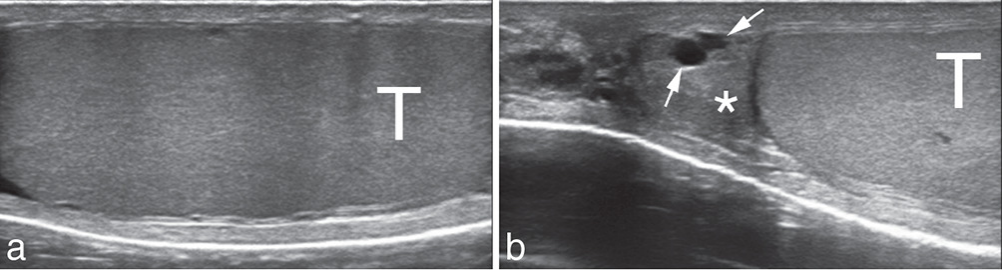
Normal appearance of (a) the left testis (T) and (b) the left epididymis (asterisk). Two small simple cysts are seen (arrows). Power Doppler evaluation (not shown) was unremarkable.

**Figure 2.**
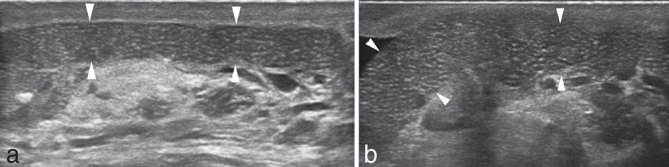
(a,b) On the external side of the left testis, an elongated, regular structure is seen (arrowheads), representing the migrated mesh. The regular, geometric echotexture can be noted. No acoustic shadow was seen. A very thin layer of fluid can be seen surrounding the mesh, although no clear signs of local inflammation can be seen. Power Doppler evaluation (not shown) was unremarkable.

The patient was sent to the radiology department for a testicular ultrasound (6–13 MHz linear high-resolution probe, MyLab™ClassC, Esaote, Genoa, Italy). Ultrasound scan revealed that the left testicle was unremarkable (Figure 1a). The epididymis had two small simple cysts (Figure 1b). No varicocele was seen. A small amount of hydrocele was seen. Colour Doppler evaluation was unremarkable. However, a tubular structure (4 cm maximum length, 1 cm thickness) with regular, geometric echotexture and tiny scattered hyperechoic spots was seen superficially on the testicle (Figure 2). No acoustic shadow was seen. This finding was compatible with the migration in the scrotum of the mesh used for previous inguinal hernia repair through the inguinal canal. A thorough ultrasound scan of the left inguinal canal failed to show the presence of an inguinal mesh. No hernia recurrence was seen.

## Treatment

As the patient was asymptomatic on admission to the hospital, no surgery was performed. The patient was discharged from the hospital with indication of abstention from physical exertion and follow-up in case symptoms recur.

## Discussion

Hernia repair is one of the most frequent operative procedures performed throughout the world.^4^ The technique has continued to evolve and the use of meshes as patches and plugs represents the standard practice.^5^

In 1999, Dieter^6^ considered mesh plug migration into the scrotum a new complication of hernia repair owing to the change in surgical technique. He reported two cases of migration, both also complicated by the presence of an incarcerated scrotal hernia, in which correction of the hernia and resection of the migrated mesh plug from the scrotum were carried out concurrently.

Nowak et al^7^ reported a case of a mesh plug positioned to treat an inguinal hernia *via* transabdominal approach during laparotomy performed in a patient operated on in emergency. Several months after surgery, the patient came back with the scrotum remarkably tender to palpation and a palpable mass at the apex of the right scrotum. At surgery, he had an incarcerated right scrotal hernia and the mesh plug represented the scrotal mass felt on pre-operative examination. The authors concluded that the indication to treat an asymptomatic scrotal hernia during emergency surgery was incorrect.^7,8^

Mesh migration into the scrotum is a rare complication of inguinal hernia repair. In a literature review, which included papers published from 1995 to 2006, Jeans et al^8^ found six cases of reported mesh plug migration in males ranging from 30 to 83 years of age. He found that three cases were due to poor surgical technique, one case was not a real migration, another was a case of wrong surgical indication, and in the last case, the patient was in overall very poor health. Only two of these cases concerned a migration of the mesh into the scrotum, both requiring surgical treatment. Jeans et al^8^ concluded that mesh plug migration after open inguinal hernia repair can be avoided if proper attention to detail is paid at the time of the initial repair.^9^

Our case seems to be different from those reported in the literature. First, the patient reported the occurrence of severe scrotal pain that spontaneously resolved. Also, no signs of inflammation were seen, neither on physical examination nor on blood test and ultrasound examination. Thus, we can hypothesize that the pain was unrelated to the scrotal mass, which was therefore discovered incidentally. Also, no clinical or ultrasound signs of recurrent hernia were seen.

We were not able to find in the literature any paper dealing with ultrasound evaluation of migrated mesh used for hernia repair. A few papers in the literature report the appearance of a mesh used for hernia repair, but only when evaluating complications of surgery. Crespi et al^10^ in 2004 reported the use of ultrasound and CT scan in the evaluation of post-operative complications of inguinal hernioplasty using prosthetic polypropylene mesh in 31 patients. They also performed an *in* *vitro* evaluation of the mesh to show the ultrasound appearance. They report that *in* *vitro* the prosthetic mesh appeared as a linear hyperechoic image measuring about 2 mm in thickness, with a posterior acoustic shadow and a finely irregular surface. *In* *vivo,* they reported a similar appearance, occasionally without the presence of the posterior acoustic shadow. In a pictorial review, Parra et al^9^ reported that ultrasound appearance is related to the composition of the mesh itself and an acoustic shadow is not always visible.

In our case, we do not have any clinical information regarding the type of surgery and the composition of the implanted mesh. The elongated tubular structure with regular geometric echotexture seen on our images may recall a rolled up thin structure (*e.g.* the mesh). No other cause of paratesticular mass may present with that appearance. The absence of signs of inflammation on ultrasound (*e.g.* negative colour Doppler evaluation, absence of fluid or oedema) suggested that the migration was not a recent event and that probably the pain reported by the patient was not related to the mesh itself.

One remarkable limitation of our report is that the patient did not undergo surgery. Thus, the only confirmation of our hypothesis is based on both the affirmation of the patient of having been implanted with an inguinal mesh and the fact that the mesh was not seen on ultrasound scan of the groin. Also, another imaging modality (*e.g.* CT scan) may have been useful to confirm the diagnosis. However, it was not considered clinically adequate as the patient was asymptomatic at that moment.

## Conclusions

We reported an unusual case of scrotal mass, which on ultrasound evaluation resembled a migrated mesh used for inguinal hernia repair. Ultrasound scan was able to depict clearly the appearance of the migrated mesh, thus avoiding surgical exploration.

## Learning points

1. A mesh used for inguinal hernia repair can migrate into the scrotum, mimicking a scrotal/testicular mass.
2. Ultrasound scan may help in detecting the mass and highlighting the typical echotexture.
3. Ultrasound scan of the inguinal canal demonstrating the absence of the mesh may help in orienting the diagnosis.

## Consent

Consent to publish was obtained.
